# Do endometrial immune changes with age prior to menopause compromise fertility in women?

**DOI:** 10.37349/ei.2022.00076

**Published:** 2022-10-29

**Authors:** Mickey V. Patel, Zheng Shen, Charles R. Wira

**Affiliations:** Department of Microbiology and Immunology, Geisel School of Medicine at Dartmouth, Lebanon, NH 03766, USA

**Keywords:** Endometrium, aging, immunity, reproduction, implantation, epithelial cells, CD8+ T cells, menopause

## Abstract

Menopause signals the end of the reproductive period in women. However, fertility and fecundity decrease with increasing age prior to menopause demonstrating that changes in the premenopausal female reproductive tract (FRT) are already occurring that negatively impact reproductive success. The effects of age on the endometrium are poorly understood, in contrast to the ovary where changes occur with increasing age that negatively affect successful reproduction. The endometrial immune system is essential for generating a receptive endometrium, but the link between the immune and reproductive systems in the endometrium in the years prior to menopause has not been well-defined. Since the endometrial immune system is tightly regulated to maximize reproductive success and pathogen protection, changes in immune function with increasing premenopausal age have the potential to impact reproduction.

## Introduction

Premenopausal women naturally undergo a gradual decline in fertility and fecundity with increasing age beginning in their mid-30s [1] through to menopause in their 50s when ovarian and reproductive function cease. Furthermore, in many developed countries, women are increasingly delaying childbirth to a later age than in previous generations [1]. These overlapping trends mean that women from their mid-30s onwards face increased difficulty in achieving reproductive success, since the desire for conception coincides with a decline in fecundity, thus leading to increased demand for assisted reproduction [2].

Considerable efforts have been made to define the biological basis for reduced reproductive success in older premenopausal women, with a particular focus on changes in ovarian function [3]. Previous studies have shown that the ovaries are not protected from the effects of aging [4]. For example, as the ovaries age, there is a decline in the total number and quality of oocytes [5], a shortening of the ovarian cycle [6], and increased secretion of follicle-stimulating hormone (FSH) [7, 8], as well as other changes [4, 9]. Together, these changes are major contributors to the decline in fertility and fecundity faced by older premenopausal women. However, reproductive success is not only determined by changes in ovarian function.

Beyond the ovary, the uterus is the other major anatomical site where many key events of reproduction occur, such as blastocyst attachment and implantation, as well as placental development. Central to this essential role is the generation of a receptive endometrium that can support implantation and pregnancy. Defects in endometrial receptivity could be a major reason for implantation and pregnancy failure as premenopausal women age. However, the changes that occur in endometrial receptivity with increasing age in premenopausal women, and whether this impacts reproductive success, are unclear.

Similar to the ovaries, the uterus also is affected by increasing age in premenopausal women which may be detrimental to successful reproduction. Women older than 45 years show increased uterine senescence compared to women below 45 years of age [10]. Recent transcriptomic studies have shown that increasing age affects multiple gene expression pathways linked to reproduction in the endometrium [11]. For example, genes linked to endometrial ciliary processes and epithelial proliferation were dysregulated with increasing premenopausal age [11]. While *in vitro* fertilization (IVF) implantation rates are equivalent between older and younger women, older women have higher rates of pregnancy loss and a greater risk of embryo-endometrium asynchrony [12–14]. Using donor oocytes from younger women, Borini et al. [15] found that pregnancy rates were higher in younger (< 39 years) *versus* older (40–49 years) premenopausal women. Since older women had lower pregnancy rates than younger women despite receiving oocytes from the same individual, this suggested that uterine age, and its accompanying phenotypic changes, may impact pregnancy success rates [15]. However, in other studies, the same group demonstrated it was possible for women between the ages of 50–62 to be successfully implanted with donor oocytes following hormone replacement therapy with estradiol and progesterone, suggesting that age-dependent changes in uterine phenotype can be partially alleviated by hormonal stimulation [16].

Animal models also demonstrate an age-dependent effect on uterine phenotype. For example, previous studies have shown a reduction in uterine wet weight, uterine glycogen content, increased post-implantation failure, and reduced decidualization in older *versus* younger mice [4, 17–23]. There is also a reduction in genes associated with cell proliferation with increasing age in mice uteri [24], while immune and inflammatory gene expression increases with age in rat uterine horns [17]. Whether aging-induced changes in the uterus exacerbate the decrease in fecundity due to impaired ovarian function, thus leading to a steeper decline in successful reproduction, is not clear.

## Endometrial immune system

The endometrial immune system is essential for reproductive success [25]. Unlike other mucosal surfaces, the pre-menopausal endometrial immune system has two contrasting tasks: (i) providing protection against infections while (ii) selectively modulating maternal immune function to create a permissive environment for reproductive success. This is apparent during the window of implantation (days 19–23) of the secretory phase of the menstrual cycle. During this period, multiple aspects of both the innate and adaptive immune systems in the endometrium are downregulated to create a receptive endometrium in preparation for the entry of sperm, and potentially, a semi-allogeneic blastocyst. These changes in immune function led to the hypothesis of a window of vulnerability (days 15–25) when women are at increased risk for viral infection, which partially overlaps with the window of implantation [26]. This was subsequently demonstrated by Kersh et al. [27] and Vishwanathan et al. [28] who found increased simian immunodeficiency virus (SIV) infection of monkeys exposed to the virus during the luteal phase compared to the follicular phase [27, 28].

A well-regulated endometrial immune response is a key ingredient for reproduction [29, 30]. Using endometrial biopsies as a means of immune profiling, several studies have demonstrated that local endometrial immunity is an important parameter that influences the outcome of pregnancy [29, 30]. Controlled endometrial inflammatory responses are essential for successful reproduction and have been linked to increased IVF success rates [31]. For example, inflammation due to endometrial biopsies leads to increased rates of successful implantation in women [32]. However, excessive inflammatory responses can negatively impact reproductive success. Activation of inflammasome-regulated pathways is hypothesized to impair endometrial receptivity in women [33, 34]. Furthermore, inflammatory responses due to pathogens in the endometrium also negatively impact reproduction [35]. As discussed below, many components of innate and adaptive immunity are integral to and shared with reproductive processes. This overlap between the immune and reproductive systems, and how the effects of aging on immunity can directly affect reproduction in pre-menopausal women, is poorly understood.

## Menopause and endometrial immune function

Menopause, which occurs at the average age of 50 years [36], is the end of the reproductive period in women and is accompanied by the permanent cessation of menstrual cycles due to the decline in ovarian sex hormone levels. This loss of cyclic hormone stimulation leads to changes in endometrial immune function across multiple cell types [37]. There are changes in both innate and adaptive immune cell numbers and function throughout the human female reproductive tract (FRT) following menopause [37–42]. For example, the number of CD11c+ endometrial dendritic cells (DCs) declines with increasing age while endometrial DCs from post-menopausal women have a greater capacity to induce CD103+ expression on CD8+ T cells than DCs from premenopausal women [43]. In contrast to the DCs, the number of CD4+ T helper type 17 (Th17) cells, as well as C-C motif chemokine receptor 5 (CCR5)+ CD4+ T cells, significantly increases in the post-menopausal endometrium and is more susceptible to human immunodeficiency virus (HIV) infection [44]. Together these studies demonstrate that loss of ovarian hormone exposure can fundamentally alter immune function in the endometrium.

Since fecundity begins to decline from the mid-30s onwards, changes in endometrial immune function may already be underway prior to menopause. As discussed below, one mechanism by which this could occur is the increased variability in sex hormone production as women enter perimenopause. Whether the endometrium undergoes a decline in reproductive potential due to changes in immunological function with increasing age in premenopausal women is unknown. Identifying the immunological changes that occur in the aging premenopausal endometrium, defining the biological mechanisms underpinning them, and understanding how these can be modulated to increase the chances for reproductive success, will be essential in future decades as women increasingly delay childbirth into their less fertile years.

## Regulation of endometrial immune function by sex hormones

In premenopausal women, the endocrine system, via the secretion of hormones and growth factors, particularly the sex hormones estradiol and progesterone, maintains tight control of the endometrial immune system [37–42]. As women approach menopause and enter perimenopause, there is increased variability in the length of the menstrual cycle and sex hormone levels [45]. Similarly, the secretion of estradiol and progesterone by the placenta begins earlier in women below 40 years compared with women above 40 years [12]. In animal models, older female mice and cows show reduced levels of progesterone compared to younger females [19, 46, 47]. Additionally, estrogen and progesterone receptor expression decline with age in rodents [48]. Together, these studies demonstrate that the production of sex hormones begins to change with increasing premenopausal age. Since sex hormones have potent effects on endometrial immune function and are the primary mechanism by which immune protection is downregulated during the window of vulnerability/implantation, changes in the concentration and duration of sex hormone exposure, or the expression of sex hormone receptors can potentially lead to alterations in immune function. This in turn could lead to increased susceptibility to incoming pathogens which can disrupt the reproductive process.

## Endometrial epithelial cells

Endometrial epithelial cells exemplify the contrasting balance between reproductive function *versus* immune protection that cells in the endometrium must accommodate [49]. They are essential mediators of both constitutive and induced innate immune protection [49, 50]. For example, throughout the menstrual cycle, antimicrobials including secretory leukocyte protease inhibitor (SLPI) and macrophage inflammatory protein 3alpha (MIP3α) are secreted into the uterine lumen where they maintain a level of baseline protection against potential pathogens [51, 52]. As the first mucosal cells exposed to potential incoming pathogens, they can mount a robust innate immune response to directly inhibit pathogen survival as well as recruit and activate immune cells. Epithelial cells are also essential for reproductive success and are the first endometrial cells to interact with the developing blastocyst and are important players in the events leading to attachment and implantation [53].

**Figure 1.**
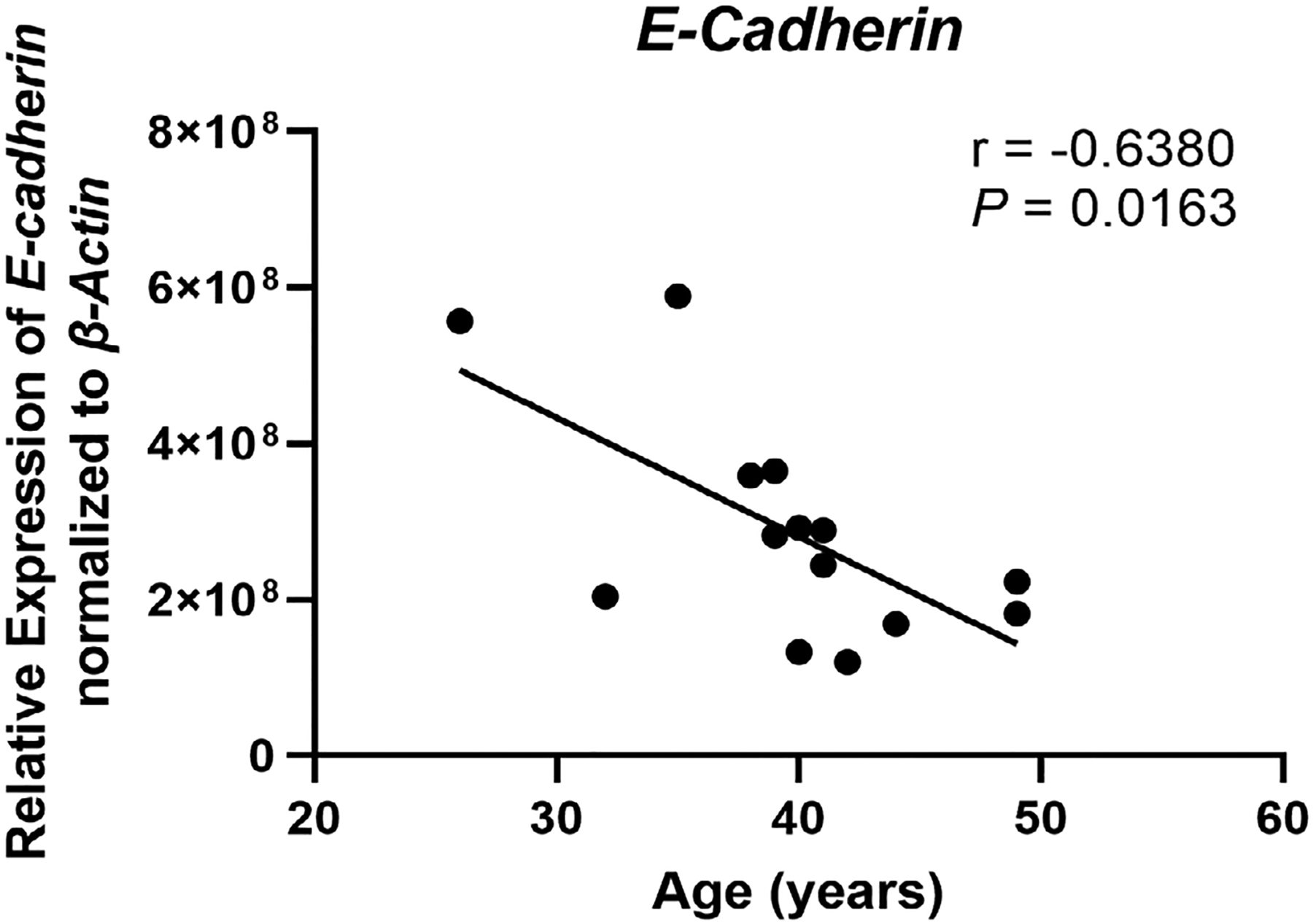
*E-cadherin* expression decreases with increasing age in premenopausal women. *E-cadherin* (CDH1) mRNA expression was determined by real-time reverse transcription-polymerase chain reaction (RT-PCR) for endometrial epithelial cells grown *in vitro* from premenopausal women (*n* = 14), isolated from either the proliferative or secretory phase, between the ages of 26–49 years. Expression is normalized to the expression of the housekeeping gene *β-Actin*. Each symbol represents an individual patient. Non-parametric Spearman correlation analysis

A key function of endometrial epithelial cells is to form a physical barrier that prevents incoming pathogens from accessing the stromal environment. Linking the columnar epithelial cells of the endometrium is a network of tight junctions and adherens junctions [54]. Previously we and others have shown that sex hormones can modulate epithelial barrier function via changes in transepithelial resistance [55]. Estradiol suppresses transepithelial resistance of endometrial epithelial cells demonstrating that sex hormones can alter the permeability of tight junction complexes [55]. Multiple pathogens including HIV are capable of disrupting the barrier function of epithelial cells in order to gain access to susceptible target cells by altering the expression of proteins that constitute these complexes [56]. These junctional complexes are also necessary for appropriate interactions between the blastocyst and epithelium as they determine cell polarity [57–59]. Disruptions to cell polarity in turn lead to decreased blastocyst attachment and implantation. E-cadherin (CDH1), a component of adherens junction complexes, is essential for embryo attachment. Previous studies have shown that knockout of E-cadherin expression in mice leads to decreased implantation [60]. In murine uterine epithelial cells, E-cadherin expression is upregulated in the peri-implantation uterus at implantation sites under the control of progesterone [61], and reduction of E-cadherin expression leads to decreased attachment and implantation [62]. E-cadherin expression is significantly lower in the endometrium of women with recurrent implantation failure and recurrent miscarriage compared to normal fertile women [63]. In recent preliminary studies, we found that messenger RNA (mRNA) expression of *E-cadherin* in endometrial epithelial cells recovered from proliferative and secretory phase premenopausal women and subsequently grown *in vitro* decreased significantly with increasing age (25–50 years) (Figure 1). This surprising observation suggests that as women age, aspects of epithelial cell barrier function decline, potentially affecting cell polarity, and possibly decreasing the likelihood of successful blastocyst attachment while also allowing increased pathogen penetration into the underlying tissues to cause inflammation which can compromise fertility. However, further studies using a larger and more diverse cohort of patients and using tissues only recovered from the secretory phase of the cycle are required to conclusively demonstrate these changes. Additionally, whether *in vitro* studies using single cells accurately reflect changes *in vivo* remain to be determined.

Given their anatomical location, epithelial cells are often the first cells exposed to incoming pathogens and thus function as sentinels of the innate immune system. Key to this is the expression of a panel of pattern recognition receptors (PRRs) including Toll-like receptors (TLRs) and RIG-like receptors (RLRs). These PRRs recognize conserved elements on foreign pathogens allowing them to detect a broad range of viral, fungal, and bacterial pathogens. Our previous studies have shown that epithelial cells express a full panel of PRRs and that in response to PRR stimulation, they mount a potent immune response characterized by increased secretion of inflammatory cytokines, chemokines, and interferons [64–67]. However, PRRs are not only mediators of the innate immune response. Studies in murine models have shown that TLRs are essential for blastocyst adhesion and implantation [68–71], and that loss of TLR expression can lead to decreased reproductive success.

**Figure 2.**
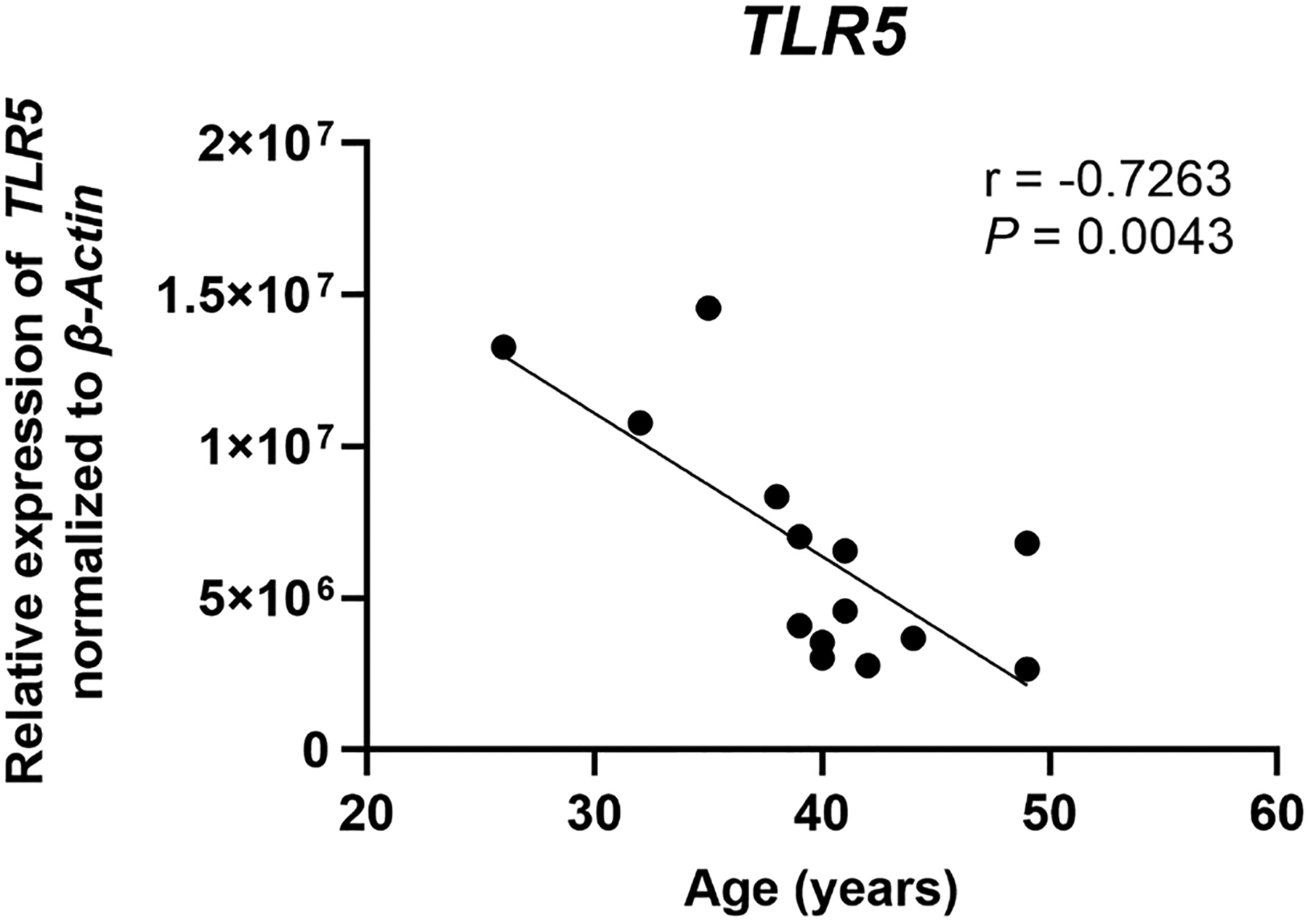
*TLR5* expression decreases with increasing age in premenopausal women. *TLR5* mRNA expression was determined by real-time RT-PCR for endometrial epithelial cells grown *in vitro* from premenopausal women (*n* = 14), isolated from either the proliferative or secretory phase, between the ages of 26–49 years. Expression is normalized to the expression of the housekeeping gene *β-Actin*. Each symbol represents an individual patient. Non-parametric Spearman correlation analysis

In humans, there have been fewer studies linking PRR expression and signaling to reproductive failure. However, similar to animal models, PRR expression has been linked to multiple reproductive processes, and that appropriate PRR signaling is essential for reproductive success [72]. Multiple PRRs have been implicated in these processes, including TLR5, a bacterial PRR. In studies utilizing endometrial biopsy samples from healthy women recovered at different stages of the cycle, TLR5 expression was shown to decrease during the window of implantation [73]. Trophoblast cells enhance the response of endometrial epithelial cell lines to flagellin, the ligand for TLR5 [74]. Furthermore, *in vitro* exposure of endometrial cell monolayers to flagellin led to reduced attachment of spheroids [75]. In preliminary studies, we analyzed the mRNA expression of TLR5 in endometrial epithelial cells grown *in vitro* from a limited number of proliferative and secretory phase premenopausal women (*n* = 14) and found that TLR5 expression decreases significantly between 25 years and 50 years of age (Figure 2). Since TLR5 is important for trophoblast adhesion, our findings provide preliminary evidence that suggests that decreases in TLR5 expression could affect the rate of successful attachment, potentially by altering the inflammatory response, and thus communication between the endometrium and trophoblast. Our findings further suggest that as premenopausal women age, recognition of foreign pathogens by the endometrial epithelium is likely to be impaired, possibly leading to increased pathogen survival that in turn compromises reproductive success.

Endometrial epithelial cells secrete a range of cytokines, chemokines, and antimicrobials including SLPI [51], human β-defensin 2 (HBD2) [55], and transforming growth factor β (TGFβ) [76] which can modulate the function of immune cells present in the endometrium. For example, TGFβ can suppress endometrial CD8+ T cell cytolytic activity [77]. The secretion of several of these proteins is under hormonal control. TGFβ secretion by endometrial epithelial cells is stimulated by progesterone [78], while SLPI and HBD2 expression is stimulated by estradiol [51, 55, 79]. Estradiol also inhibits interleukin-1β (IL-1β)-mediated proinflammatory responses, which are important in implantation [80], by endometrial epithelial cells [81]. Whether changes in sex hormone levels that occur as women enter perimenopause affect the epithelial expression of these proteins is unknown.

**Figure 3.**
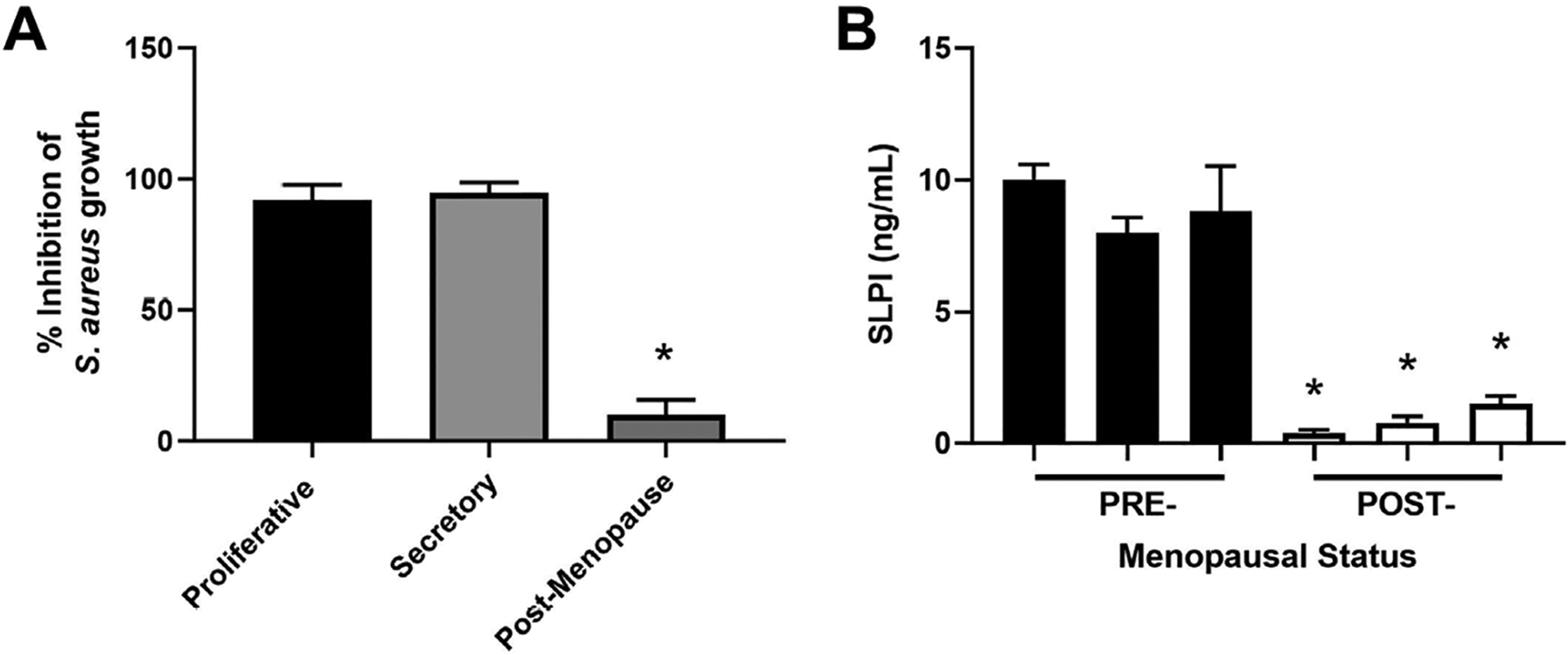
Antimicrobial activity and SLPI secretions by endometrial epithelial cells decrease following menopause. (A) *S. aureus* was incubated with apical secretions from polarized endometrial epithelial cells isolated from the proliferative (*n* = 6), secretory (*n* = 6), and post-menopausal (*n* = 4) stages and bacterial growth subsequently determined; (B) apical secretions from pre- (*n* = 3) and post- (*n* = 3) menopausal women were analyzed for SLPI expression by enzyme-linked immunosorbent assay (ELISA). Data is shown as mean ± SEM. * *P* < 0.05; SEM: standard error of the mean. Data is derived from Fahey et al. [51]

The secretion of antimicrobials by epithelial cells is a key component of immune protection against pathogens since infections such as *Staphylococcus aureus* (*S. aureus*) can contribute to infertility and reproductive failure [82–84]. To measure antimicrobial activity, we incubated *S. aureus* with apical secretions from polarized endometrial epithelial cells from pre- and post-menopausal women (Figure 3) [51]. Unlike apical secretions from postmenopausal women which had low antibacterial activity, premenopausal secretions inhibited *S. aureus* colony formation by 90% [51]. In other studies, endometrial epithelial secretions significantly inhibited *Neisseria gonorrhoeae* (*N. gonorrhoeae*), HIV-1, and *Candida albicans* (*C. albicans*) without affecting *Lactobacillus crispatus* (*L. crispatus*), a part of the normal vaginal microflora [85]. To determine whether antimicrobial levels change with menopausal status (Figure 3), we measured SLPI in apical secretions from endometrial epithelial cells and found that while SLPI was present in premenopausal secretions, it was barely detectable in postmenopausal secretions. Antibody neutralization of SLPI reduced antibacterial activity in premenopausal secretions by 50% [51]. As a part of these studies, we found that incubation of primary polarized endometrial cells with estradiol increased SLPI secretion and HBD2 mRNA expression as well as enhanced antibacterial activity against *S. aureus* [51, 55, 79]. Whether SLPI and other hormonally regulated antimicrobials decline before menopause given the changes in circulating estradiol during perimenopause remains to be determined. Changes in the levels of these protective molecules may lead to increased infiltration of pathogens that could lead to excessive immune activation and thus negatively impact endometrial receptivity.

Beyond their role in immune protection, antimicrobials such as SLPI and elafin are also essential for endometrial tissue remodeling that occurs in the secretory phase during decidualization in preparation for implantation, as well as during pregnancy to optimize conditions for fetal development. Changes in the levels of these proteins could therefore directly affect the generation of a receptive endometrium.

### CD8+ T cells

Leukocytes account for 6–20% of cells from the upper and lower FRT in pre-menopausal women [86]. Most of these cells consist of T-lymphocytes, including CD3+ lymphocytes. Within the CD3+ population, CD8+ T cells account for approximately 50% of those present in the endometrium [86, 87]. In contrast to those in the endocervix, endometrial CD8+ T cells are CCR5+ and have an effector-memory phenotype [88]. Previous studies from our laboratory indicate that CD8+ T cells are hormonally regulated during the menstrual cycle [89, 90]. For example, while overall CD8+ T cell numbers remain relatively stable across the menstrual cycle, their distribution within human endometrial tissue varies considerably. CD8+ T cells are present in lymphoid aggregates which increase in size during the proliferative phase and reach maximal size during the secretory phase of the menstrual cycle [89, 90]. Following menopause, these structures are absent in the endometrium, providing evidence that lymphoid aggregates are under hormonal control. In contrast to the endometrium, lymphoid aggregates are not present in human endocervical or ectocervical tissue [89, 90].

**Figure 4.**
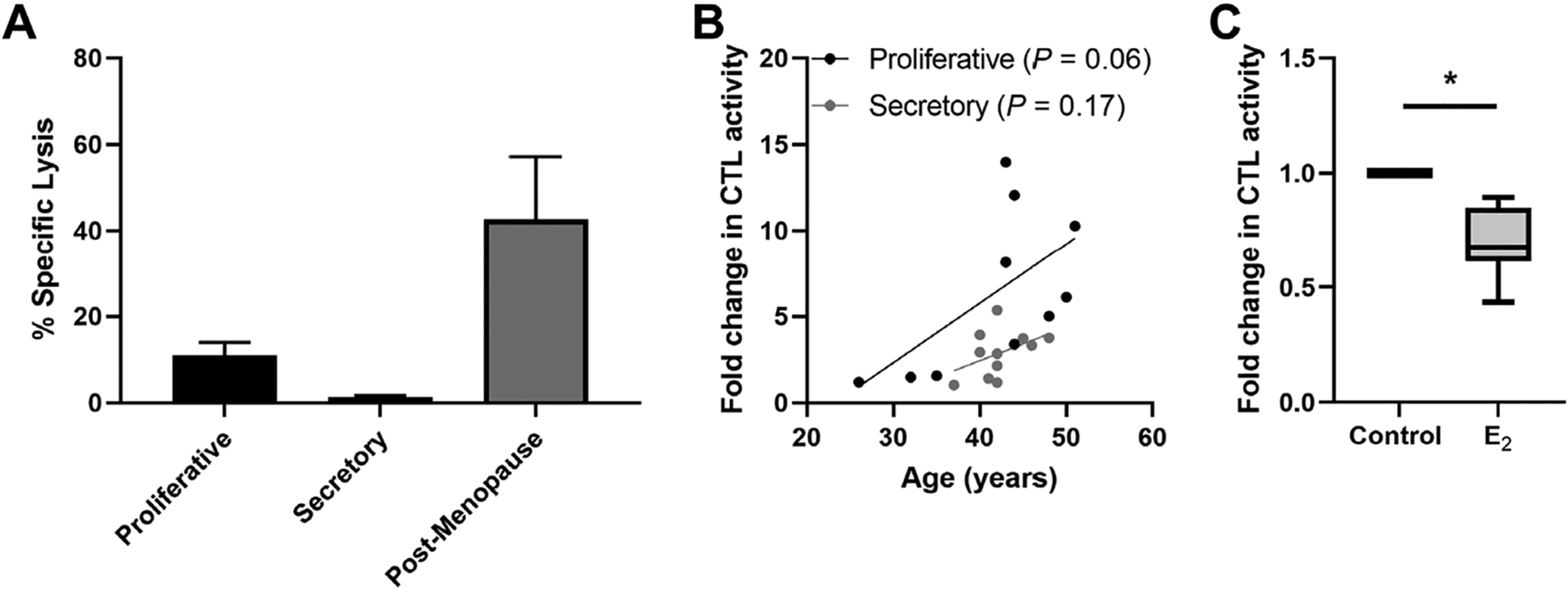
Endometrial CD8+ T cell cytotoxic killing is suppressed in the secretory phase of the menstrual cycle. (A) Redirected lysis assay to measure cytotoxic activity in endometrial mixed cell suspensions showing decreased killing in proliferative and secretory phase and increased killing in postmenopause. Data is derived from White et al. [93]; (B) direct endometrial CD8+ T cell CTL activity trends upwards with increasing age in both the proliferative and secretory phase in pre-menopausal women; (C) 17-β estradiol (E_2_; 5 × 10^−8^ mol/L; 48 h) treatment of endometrial CD8+ T cells suppresses their CTL activity. * *P* < 0.05. Data in B and C is derived from Shen et al. [78]

Beyond their distribution, sex hormones can directly modulate CD8+ T cell function including cytotoxic T lymphocyte (CTL) activity. Endometrial CD8+ T cell cytotoxic killing is significantly suppressed following exposure to estradiol (Figure 4) [78]. Furthermore, progesterone treatment of endometrial epithelial cells leads to the upregulation of TGFβ which in turn suppresses CD8+ T cell cytotoxic killing [78]. As discussed elsewhere, hormonal suppression of CD8+ T cell cytotoxic killing is likely to optimize conditions for successful implantation of a semi-allogeneic blastocyst [26].

**Figure 5.**
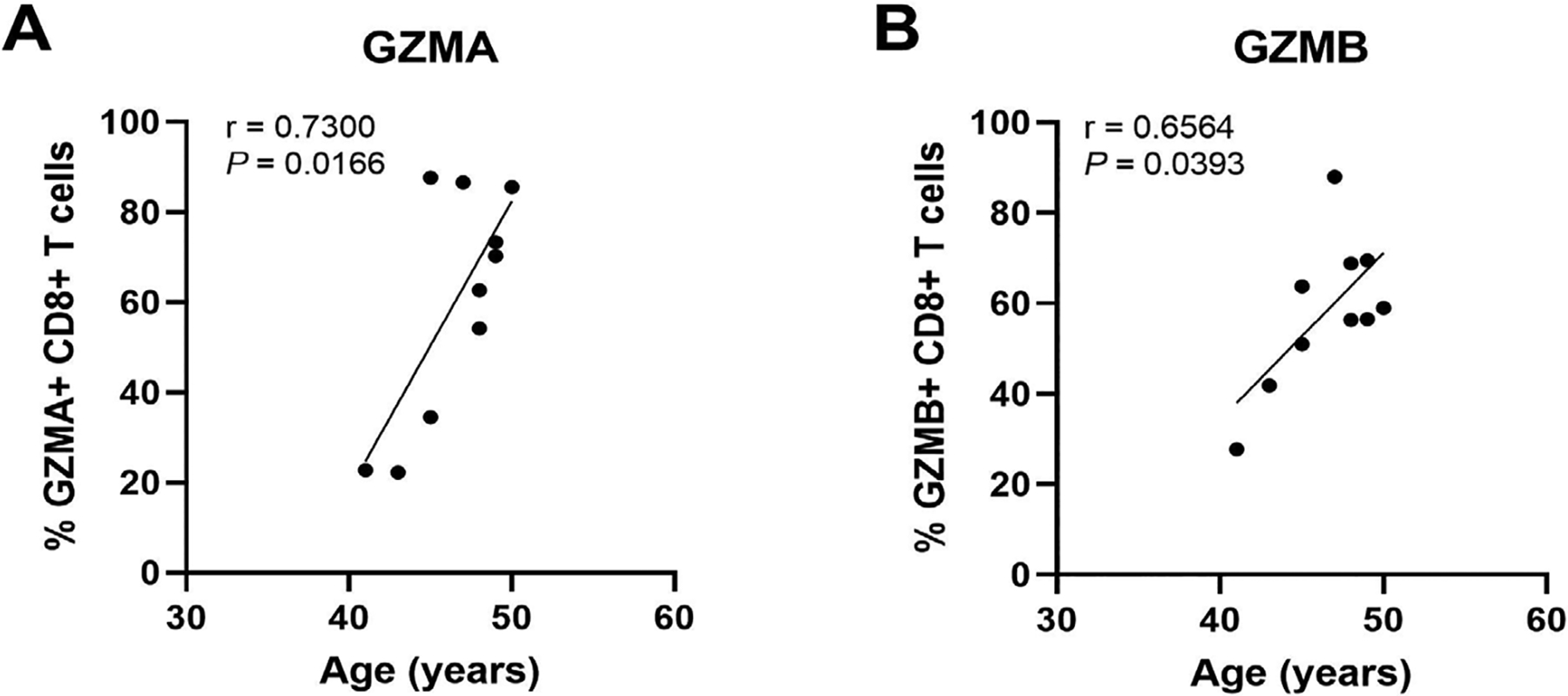
The percent of GZMA+ and GZMB+ endometrial CD8+ T cells increases with increasing age in pre-menopausal women. Endometrial CD8+ T cells in mixed cell suspensions were analyzed for intracellular GZMA and GZMB by flow cytometry. Each point represents a single individual (*n* = 10). Non-parametric Spearman correlation analysis. Data is derived from Shen et al. [112]

Regulation of endometrial CD8+ T cells, which provide protection through cell-mediated killing, is essential for successful reproduction since the suppression of CD8+ T cell killing is necessary for maintaining a tolerogenic environment [91]. Several studies suggest that altered functions of memory CD8+ T cells are linked to recurrent miscarriages [92]. Building on our earlier studies, we found that endometrial CD8+ cytotoxic killing significantly decreases between the proliferative and secretory phases of the menstrual cycle (Figure 4) [87, 93, 94], indicating that downregulation of cytotoxic capacity is important for the early stages of reproduction. As a part of these studies, cytotoxic killing capacity by tissue-resident CD103+CD8+ T cells was significantly lower than non-resident CD103–CD8+ T cells. Following menopause endometrial CD8+ T cell cytotoxic killing significantly increases, along with a concomitant rise in the expression of key cytotoxic proteins such as perforin and granzyme A (GZMA) and B (GZMB), compared to the premenopausal endometrium [87, 93]. We investigated whether changes in endometrial CD8+ T cell cytotoxic killing varied between younger and older premenopausal women and unexpectedly found that endometrial CD8+ T cell killing increases with age in premenopausal women in the years leading up to menopause (Figure 4). More recently we found that the number of GZMA+ and GZMB+ endometrial CD8+ T cells increases between 40 and 50 years of age (Figure 5) (Shen et al. submitted 2022). Overall, these findings suggest that increases in CTL activity during the menstrual cycle can lead to reduced reproductive success and demonstrate that changes in adaptive immunity are already occurring in the decade prior to menopause. Other studies have shown that decidual CD8+ T cells have tissue-residency phenotype (CD103+) and that they express lower levels of granzyme and perforin than CD103− cells [95]. Most endometrial CD8+ T cells during the window of implantation are primarily CD69+ and CD103+ which are markers of tissue residency [92]. Decreased CD69 expression is associated with increased rates of recurrent pregnancy loss [92]. This strongly suggests that suppression of cytotoxic killing capacity is needed for a successful pregnancy. Further studies are needed to identify the underlying mechanisms responsible for increasing cytotoxic killing prior to menopause. What is clear is that CD8+ T cells are primary candidates for decreased reproductive success.

## Other immune and non-immune cells

Successful reproduction requires appropriate regulation of multiple cell types beyond epithelial cells and CD8+ T cells. Other cell types are essential for reproduction, and it is likely that the combined effects of all cell types create a receptive endometrium. Two other cell types that have crucial roles in successful reproduction are endometrial natural killer (NK) cells and fibroblasts.

Uterine NK cells are distinct from those in the blood and the rest of the FRT. Previous studies have shown that they are the most abundant immune cells in the secretory endometrium [41]. Their proliferation during the secretory phase is partially regulated by endometrial fibroblasts via the secretion of IL-15, which is under the control of progesterone [96]. While the effect of aging on uterine NK cells is relatively unknown, several studies have shown that aging affects the phenotype and distribution of blood NK cells [97]. For example, the proliferation capacity of blood NK cells is reduced with age [98], though they retain their capacity to respond to sex hormones [99]. Furthermore, NK cells from younger women upregulate the expression of interferon-γ (IFNγ), MIP1-α, and IL-8 to a greater extent than those from older women [98, 100–104]. Together these studies suggest that uterine NK cells may undergo phenotypic changes with increasing premenopausal age that in turn affect the generation of a receptive endometrium.

Fibroblasts undergo decidualization during the secretory phase of the menstrual cycle in preparation for possible implantation and are a necessary component of a receptive endometrium. Recent *in vitro* studies have shown that age negatively affects endometrial stromal cell proliferation with significantly reduced proliferation in cells from older (36–46 years) *versus* younger (25–35 years) women [105]. In addition, these cells have reduced mRNA expression of bone morphogenetic protein 2 (BMP-2) and signal transducer and activator of transcription 3 (STAT3), as well as the decidualization markers prolactin (PRL) and insulin-like growth factor binding protein-1 (IGFBP-1) [105]. Similar results have been obtained showing the reduced proliferative capacity of murine endometrial stromal cells. Together these studies suggest that the ability of endometrial fibroblasts to create a decidualized endometrium may be compromised with increasing age.

## Infections and immune dysregulation

Sexually transmitted infections (STIs) are linked with reduced fertility. *N. gonorrhoeae* and *Chlamydia trachomatis* (*C. trachomatis*) can induce tubal inflammation leading to infertility. *C. trachomatis* infections have also been linked to pelvic inflammatory disease (PID) which in turn increases the risk of infertility. Even in women with no visible tubal pathology, *C. trachomatis* infection reduces fertility and the possibility of successful pregnancy [106]. Furthermore, the implantation rate following IVF was significantly lower in women with a previous chlamydial infection [107]. HIV infection is also associated with an overall reduction in fertility for all women [108]. Whether these effects on fertility are exacerbated with aging is unknown. Progression from HIV to acquired immunodeficiency syndrome (AIDS) was linked to an even greater reduction in fertility [108]. Furthermore, in HIV-positive women, there is a slight but significant reduction in fertility with increasing age compared to HIV-negative women [109]. Whether reduced epithelial TLR expression in the endometrium of older premenopausal women contributes to the damaging effects of STIs on fertility remains to be determined.

Intriguingly, humoral immune deficiencies have not been linked to severe defects in reproductive success. Women with common variable immune deficiency (CVID) had lower fertility and the same rate of pregnancy loss as the general population in the USA [110]. However, another study showed no differences between the CVID and non-CVID populations [111].

## Conclusions

A major gap in our knowledge is that we do not know the full extent of the changes in the endometrial immune environment with increasing age in premenopausal women, particularly during the secretory phase of the menstrual cycle, and the extent to which these changes are potentially linked to reduced reproductive success in both healthy women and women with reproductive difficulties. While there is a clear difference between the pre- and post-menopausal endometrial environment, preliminary evidence suggests that menopause is not the time when some of these changes occur, but rather that transitions in both innate and adaptive immunity begin in the years prior to menopause. Further studies are needed to determine the functional changes that other immune cells (NK cells, granulocytes, DCs) and non-immune cells (fibroblasts, endothelial cells) in the endometrium undergo as a function of age prior to menopause. Furthermore, most research has been understandably performed on women confronted with reproductive difficulties. Whether changes in these women are representative of those in the healthy population is not clear, since fecundity decreases in all women with increasing age. It is important for future studies to recognize that endometrial immune and reproductive functions are not distinct, but rather two tightly interlocked systems, and that changes in immune function that occur with increasing premenopausal age will likely have significant effects on reproduction.

## Data Availability

Data and materials are available upon reasonable request (Charles R. Wira, Charles.R.Wira@Dartmouth.edu).

## Abbreviations

C. trachomatis: Chlamydia trachomatis
CTL: cytotoxic T lymphocyte
CVID: common variable immune deficiency
DCs: dendritic cells
FRT: female reproductive tract
GZMA: granzyme A
GZMB: granzyme B
HBD2: human β-defensin 2
HIV: human immunodeficiency virus
IL-1β: interleukin-1β
IVF: *in vitro* fertilization
mRNA: messenger RNA
NK: natural killer
PRRs: pattern recognition receptors
S. aureus: Staphylococcus aureus
SLPI: secretory leukocyte protease inhibitor
TGFβ: transforming growth factor β
TLRs: Toll-like receptors
